# Effect of Melatonin Administration on Mitochondrial Activity and Oxidative Stress Markers in Patients with Parkinson's Disease

**DOI:** 10.1155/2021/5577541

**Published:** 2021-10-18

**Authors:** Alicia Jiménez-Delgado, Genaro Gabriel Ortiz, Daniela L. Delgado-Lara, Hector Alberto González-Usigli, Luis Javier González-Ortiz, Margarita Cid-Hernández, José Antonio Cruz-Serrano, Fermín Paul Pacheco-Moisés

**Affiliations:** ^1^Department of Chemistry, University Center of Exact Sciences and Engineering, University of Guadalajara, Guadalajara, Jalisco, Mexico; ^2^Department of Philosophical and Methodological Disciplines, University Center of Health Sciences, University of Guadalajara, Guadalajara, Mexico; ^3^Department of Neurology, Sub-Specialty Medical Unit, Western National Medical Center, Mexican Institute of Social Security, Guadalajara, Jalisco, Mexico; ^4^Kurago Biotek, Guadalajara, Jalisco, Mexico

## Abstract

Mitochondrial dysfunction and oxidative stress are extensively linked to Parkinson's disease (PD) pathogenesis. Melatonin is a pleiotropic molecule with antioxidant and neuroprotective effects. The aim of this study was to evaluate the effect of melatonin on oxidative stress markers, mitochondrial complex 1 activity, and mitochondrial respiratory control ratio in patients with PD. A double-blind, cross-over, placebo-controlled randomized clinical trial study was conducted in 26 patients who received either 25 mg of melatonin or placebo at noon and 30 min before bedtime for three months. At the end of the trial, in patients who received melatonin, we detected a significant diminution of lipoperoxides, nitric oxide metabolites, and carbonyl groups in plasma samples from PD patients compared with the placebo group. Conversely, catalase activity was increased significantly in comparison with the placebo group. Compared with the placebo group, the melatonin group showed significant increases of mitochondrial complex 1 activity and respiratory control ratio. The fluidity of the membranes was similar in the melatonin group and the placebo group at baseline and after three months of treatment. In conclusion, melatonin administration was effective in reducing the levels of oxidative stress markers and restoring the rate of complex I activity and respiratory control ratio without modifying membrane fluidity. This suggests that melatonin could play a role in the treatment of PD.

## 1. Introduction

Parkinson's disease (PD) is a neurodegenerative disorder of unknown etiology, characterized by the loss of nigrostriatal dopaminergic neurons, which lowers dopamine levels in the striatum and leads to a movement disorder. Mitochondrial dysfunction, increased levels of oxidative stress markers, *α*-synuclein protein aggregation, and inflammation are extensively linked with PD pathogenesis [1]. In this regard, *α*-synuclein protein is capable of interacting with mitochondria, which decreases the activity of the mitochondrial enzyme complex I and significantly increases the production of reactive oxygen species. It has been suggested that mitochondrial dysfunction in nigrostriatal neurons is an event that precedes neuronal death [2].

Currently, the use of molecules with antioxidant activity such as melatonin has been proposed for the treatment of PD. Melatonin is a pleiotropic molecule produced in the pineal gland and other tissues and is involved in multiple physiological functions such as the control of circadian rhythms, anti-inflammatory properties, mitochondrial biogenesis, and energy metabolism, among others [3, 4]. Melatonin performs various antioxidant functions in the neuron, such as a scavenger of free radicals, and has the following characteristics: (a) it can be transported to different tissues in the body; (b) it is a broad-spectrum antioxidant; (c) it is transported across cell membranes; (d) its metabolites still have antioxidant properties [5]. Melatonin is mainly synthesized in the mitochondria and has been shown in animal models to increase mitochondrial activity by increasing the activity of respiratory complexes and ATP synthesis [6]. Previously, we found that melatonin treatment decreases the activity of cyclooxygenase 2, nitric oxide metabolites, and lipoperoxide levels in PD patients [7].

Proton-translocating NADH: quinone oxidoreductase (complex I) is a very large enzyme catalyzing the first step (electron transfer from NADH to coenzyme Q (CoQ)) of the mitochondrial electron transport chain. Interestingly, dysfunctions of complex I are attributed to decreased catalytic activity and/or increased production of reactive oxygen species [8]. This may cause disturbances in the respiratory control ratio (RCR). The RCR is a widely used parameter of mitochondrial function and indicates the coupling between the electron transport system and oxidative phosphorylation. Thus, high RCR indicates good function, and low RCR usually indicates dysfunction [9]. The aim of this work is to study the effect of melatonin supplementation on oxidative stress markers in plasma and mitochondrial activity (particularly, RCR and complex I enzymatic activity) and membrane fluidity in platelets of PD patients. Platelets have been used as a model for neurodegenerative diseases such as schizophrenia, PD, and Alzheimer's disease because evidence has been found that they produce neurotransmitters and contain proteins associated with neurons [10].

## 2. Materials and Methods

### 2.1. Study Design

A placebo-controlled, cross-over, randomized, double-blinded clinical trial was performed at the Movement Disorders Clinic of the Neurology Department of the Western National Medical Center, Mexican Institute of Social Security in Guadalajara, Jalisco, Mexico. This study was performed according to the updated Declaration of Helsinki, and all procedures were approved by the Ethics and Health Research Committee of the Mexican Social Security Institute (Protocol number: R-2018-785-019). The selected patients had stages 1–3 PD based on the Hoehn and Yahr scale, were more than 20 years old, and agreed to sign the informed consent letter. Excluded were patients who had movement disorders other than PD, those with previous thalamotomy, pallidotomy, or deep brain stimulation; pregnant females; and use of alcohol, coffee, or any antioxidant supplement. The design of the study has been previously described [11].

Melatonin and placebo were administered in a pharmaceutical gel form packet provided by the company Kurago Biotek®. The pharmaceutical gels were identical in appearance and packaging. Participants reported daily consumption of the supplement in a consumption publication sheet. The researchers were blinded to treatment until the study was complete.

Patients were divided into two groups using random generator software: the melatonin-placebo group and the placebo-melatonin group. The melatonin-placebo group received 25 mg melatonin at noon and 30 minutes before bedtime for three months, followed for four days without treatment (washout period), and then received 25 mg of placebo at noon and 30 min before bed for three months. The placebo-melatonin group received initial placebo during 3 months followed by a washout period and then received melatonin. This melatonin administration dosage and schedule were used in a previous clinical trial of our research group in which the expression of two clock genes (PER1 and BMAL1) were assessed and in which no adverse effects were observed except daytime sleepiness and nighttime problems [11]. Additionally, a control group of thirty clinically healthy individuals was also included to compare the baseline values of the oxidative stress markers and enzymatic activity analyzed in this study.

### 2.2. Biochemical Assays

Peripheral venous blood was obtained by venipuncture from all study participants after an 8 h overnight fast and collected in Vacutainer® polypropylene tubes (Becton Dickinson, Franklin Lakes, NJ, USA) containing ethylenediaminetetraacetic acid. Blood samples were centrifuged for 10 minutes at 1800 rpm at 4°C. The plasma and erythrocytes were separated immediately. The plasma was centrifuged at 3500 rpm for 15 minutes, and the supernatant was removed. The platelets were resuspended in KME buffer (20 mM (3-(N-morpholino) propanesulfonic acid)) (pH 7.2), 120 mM KCl, and 1 mM ethylene glycol tetraacetic acid (EGTA). Protein determination was carried out by the method of Lowry et al., using bovine serum albumin (BSA) as a standard [12].

Lipoperoxides (malondialdehyde plus 4-hydroxyalkenals) were measured by a colorimetric method using an assay kit (FR12) from Oxford Biomedical Research Inc. (Oxford, MI, USA) following the manufacturer's instructions.

Carbonyl groups in proteins were quantified in plasma using the reaction with 2,4-dinitrophenylhydrazine as described by Levine et al. [13].

Nitric oxide metabolites were determined in plasma according to [14] with minor modifications. Briefly, 400 *μ*L of plasma was added 6 mg of zinc sulfate and vortexed. Then, the samples were centrifuged at 10,000 rpm at 4°C for 10 minutes. To the resultant, supernatant was added 100 *μ*L of vanadium chloride (8 mg/mL). To reduce the NO_3_^−^ to NO_2_^−^, Griess reagent (comprising 50 *μ*L of 2% sulfanilamide and 50 *μ*L of 0.1% N-(1-naphthyl) ethylenediamine dihydrochloride) was added. Following incubation for 30 minutes at 37°C, the absorbance was read at 540 nm.

Catalase activity was assessed in 1 mL of reaction medium containing 65 *μ*M H_2_O_2_, 60 mM potassium phosphate buffer (pH 7.4) at 37°C, and 100 *μ*L plasma as described elsewhere [15].

For the enzymatic activity of the mitochondrial complex I activity quantification, platelets were lysed by sonic oscillation in a Labsonic U Braun sonicator for 20 seconds and the quantification was carried out as described elsewhere [16]. In brief, 50 *μ*L of samples was incubated at 37°C for 3 min in the reaction medium containing 25 mM of potassium phosphate, 3.5 g/L of BSA, 60 *μ*M of 2,6 dichlorophenolindophenol (DCPIP), 70 *μ*M of decylubiquinone, and 1 *μ*M of antimycin A. Afterwards, 20 *μ*L of a solution containing 10 mM of nicotinamide adenine dinucleotide, 50 *μ*L of BSA (70 g/L), and 5 mM of potassium phosphate (pH 7.4) was added. The absorbance at 600 nm was then recorded every 30 seconds for 5 minutes. Subsequently, rotenone was added and the absorbance was recorded as above. The reduction speed of the DCPIP was determined considering its molar extinction coefficient of 21.3 mM^−1^ cm^−1^.

Mitochondrial oxygen uptake was measured using a Clark-type O_2_ (Oxytherm System, Hansatech Instruments, Norfolk, England) electrode at 30°C in an air-saturated medium as reported previously with minor modifications [17]. The reaction medium (1 mL) contained 130 mM KCl, 25 mM 4-(2-hydroxyethyl)-1-piperazineethanesulfonic acid, 0.1 mM EGTA, 3 mM MgCl_2_, and 10 mM potassium phosphate (pH 7.4). Respiration in state 3 was measured after the addition of adenosine diphosphate (250 *μ*M) 2 min after preincubating the platelets. State 4 oxygen consumption was determined in the presence of the specific ATP synthase oligomycin inhibitor (8 *μ*g/mg protein). Then, the respiratory control ratio (state 3/state 2) was calculated.

The fluidity of the membranes was determined in platelets via the incorporation of the fluorescent dye 1,3 dipyrylpropane (DiPP) as reported previously. Membrane fluidity was expressed as excimer/monomer fluorescence ratio (Ie/Im), and high Ie/Im ratio indicates high membrane fluidity [18].

### 2.3. Statistical Analysis

Statistical analysis was performed with the GraphPad Prism v8.0.1 software. Data are expressed as means ± SD. Statistical significance was assessed using the one-way ANOVA test and followed by post hoc multiple comparison tests using Bonferroni correction. Differences were considered statistically significant at *p* 0.05.

## 3. Results

A detailed description of the clinical and sociodemographic characteristics of patients included in this study was previously described [11]. No serious adverse drug reactions were observed with melatonin at the doses used during the trial and were mild and transitory. Accordingly, melatonin is a molecule with an uncommonly high safety profile [19, 20].

**Figure 1:**
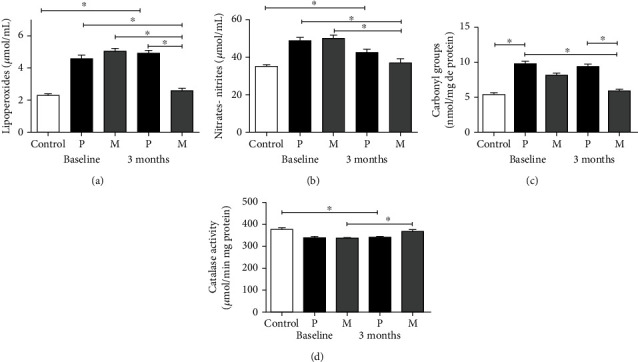
Plasma levels of oxidative stress markers at baseline and after 3 months of treatment in the placebo and melatonin groups. (a) Lipoperoxides (malonaldehyde + 4 hydroxyalkenes), (b) nitric oxide metabolites (nitrates and nitrites), (c) carbonyl groups in proteins, and (d) catalase enzyme activity. Data of the mean ± standard error and a *p* < 0.05 are shown.

At baseline, plasma levels of lipoperoxides, nitric oxide metabolites, and carbonyl groups in proteins were significantly higher in PD patients than in the healthy control group (Figures 1(a)–1(c), respectively). Conversely, the plasma activity of catalase was lower in the healthy control group than in PD patients (Figure 1(d)). These data suggest the existence of an active, persistent oxidative stress in PD. After three months of treatment with melatonin, the levels of lipoperoxides, nitric oxide metabolites, and carbonyl groups in proteins were lower than in the placebo group and were statistically similar to the levels of healthy controls. The activity of catalase was increased with the treatment with melatonin at levels similar to the control group.

**Figure 2:**
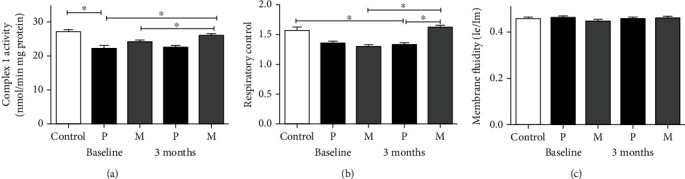
Mitochondrial parameters at baseline and after 3 months of treatment in the placebo and melatonin groups. (a) Mitochondrial complex 1 enzyme activity as measured by the oxidation of NADH, (b) respiratory control ratio, and (c) membrane fluidity. Data of the mean ± standard error and a *p* < 0.05 are shown.

At baseline, the activity of mitochondrial complex I and the respiratory control ratio were significantly lower in PD patients than in the healthy control group (Figures 2(a) and 2(b), respectively). Compared with the placebo group, the melatonin group showed significant increases of both parameters after 3 months and reached values similar to the healthy control group.

The fluidity of the membranes was similar in the melatonin group and the placebo group at baseline and after three months of treatment and was similar to the control group (Figure 2(c)).

## 4. Discussion

The results of our double-blind, cross-over trial suggest the existence of an active, persistent oxidative stress status in PD that is linked to lower mitochondrial complex I activity in platelets. These data are in consonance with previously reported data in platelets [21, 22], muscle biopsy [23], and *substantia nigra* [24]. Free radicals are by-products of the mitochondrial respiratory chain and at low concentrations are involved in homeostasis and normal cell signaling. However, increased generation of reactive oxygen species is linked to PD and complex I is one of the main sites of electron leakage to oxygen which leads to the production of the superoxide anion [1, 25]. Furthermore, the assembly of mitochondrial supercomplexes is highly susceptible to oxidative stress. For example, oxidation of phospholipids (particularly, cardiolipin) induces the disaggregation of the supercomplex formed by complex I and complex III, loss of facilitated CoQ channeling, decreased ATP synthesis [26], increased production of reactive oxygen species [27], and favors the release of cytochrome c to cytosol leading to apoptosis [28]. Furthermore, the ratio of reduced CoQ to oxidized CoQ and the ratio of reduced CoQ to total CoQ were decreased significantly in *novo* PD patients [29]. Interestingly, oxidation of cardiolipin in the *substantia nigra* is enhanced by rotenone, an inhibitor of complex I, in a model of PD [30]. Therefore, it can be expected that inhibition of cardiolipin oxidation allows a correct functioning of the mitochondria. Accordingly, as shown in a model of PD, adequate levels of cardiolipin are crucial for efficient electron transport between CoQ and complex [31] and to maintain normal mitochondrial cristae structure and correct assembly of the electron chain supercomplexes [32].

Intervention with daily supplementation of 50 mg of melatonin, for three months, resulted in a significant reduction of oxidative stress markers. These data are according to the reported previously [6] and were paralleled with significant increases of catalase, complex I activity, and respiratory control ratio. In consonance, previous data showed that melatonin increases the levels of reduced glutathione [33], decreases malondialdehyde levels, and stimulates gene expression of important antioxidant enzymes such as superoxide dismutase, complex I, and catalase [34, 35] in rat models of PD. In addition, melatonin prevents cardiolipin loss and oxidation which avoids mitochondrial membrane permeabilization induced by reactive oxygen species and other factors [36]. Reduced glutathione levels are increased by melatonin action, and glutathione also contributes to maintain the correct mitochondrial redox status and the integrity of the mitochondrial membranes [37]. Melatonin also has anti-inflammatory effects by diminishing cyclooxygenase type 2 activity in PD patients [6] and in MPTP-induced PD in mice [38]. Additionally, melatonin lowers the activation of inducible nitric oxide synthase, a well-known pathological marker of neuroinflammation [39, 40], and also decreases protein lipase A2, lipoxygenase, and cytokine activities owing to its antioxidant actions [41]. Therefore, nitrosative stress and inflammation are diminished by the action of melatonin.

Herein, we find that administration of melatonin is capable of diminishing oxidative stress markers and restoring the enzymatic activity of complex I and the coupling between electron transport and phosphorylation (ATP synthesis) processes (i.e., the RCR). Interestingly, membrane fluidity was not modified by melatonin treatment. Consistent with this proposal, melatonin treatment prevented the loss of the integrity and function of the striatal mitochondria in a chronic model of PD by preserving the normal levels of ATP and mitochondrial respiration [26, 42], and the loss of the mitochondrial membrane potential that may trigger the activation of the permeability transition pore [43]. Furthermore, melatonin significantly decreased neuronal death and mitochondrial fragmentation in an *in vitro* model of PD [44, 45]. Interestingly, it has been proposed that melatonin physically interacts with complex I at its amphipathic ramp close to the site of electron leakage: the iron-sulfur cluster N_2_ [46], reverses the decrease in mitochondrial complex 1 activity that is induced by toxins such as 1-methyl-4-phenyl-1,2,3,6-tetrahydropyridine [47], and upregulates the expression levels of subunits 1, 3 [48] ND1, ND2, ND4, and ND4L of complex I [49].

Taken together, our data showed that melatonin supplementation recovers mitochondrial function and diminishes oxidative stress. Thus, this indolamine could play a role as an adjuvant in the treatment of PD.

PD is a very complex syndrome, and there are multiple interactions of crucial phenomena such as intracellular mitochondrial dynamics, altered protein degradation, mitochondrial dysfunction, *α*-synuclein aggregation, calcium homeostasis, and impaired neurotransmitter function. Accordingly to that, a complete molecular map has been proposed that shows all the pathways involved in PD and covers everything from genes, molecules, and cells to metabolic alterations [50]. Considering the above, the limitations of our study were the lack of measurements of the effects of melatonin on some of these phenomena. However, our intention was to evaluate a small part of the mitochondrial defects associated with PD.

## Data Availability

Data are available upon request.
